# miR-155 and miR-122 Expression of Spermatozoa in Obese Subjects

**DOI:** 10.3389/fgene.2018.00175

**Published:** 2018-05-29

**Authors:** Paulina López, Andrea Castro, Martha Flórez, Karen Miranda, Pilar Aranda, Cristina Sánchez-González, Juan Llopis, Miguel Arredondo

**Affiliations:** ^1^Micronutrient Laboratory, Institute of Nutrition and Food Technology, University of Chile, Santiago, Chile; ^2^Institute of Maternal and Child Research, Faculty of Medicine, University of Chile, Santiago, Chile; ^3^CIBM, INYTA, IMUDS, Department of Physiology, Faculty of Pharmacy, University of Granada, Granada, Spain

**Keywords:** obesity, spermatozoa, pro-inflammatory cytokines, iron, microRNAs

## Abstract

Obesity is characterized by mild chronic inflammation that is linked with impaired iron homeostasis. Studies in human and murine show that there is a transgenerational epigenetic inheritance via the gametes in obesity; however, there is little information on changes in the expression of microRNAs related to inflammation and iron homeostasis in spermatozoa from obese subjects. The present study investigated the expression of microRNAs related to inflammation (miR-21 y miR-155) and iron nutrition (miR-122 and miR-200b) in plasma, peripheral blood mononuclear cells (PBMC) and spermatozoa from normozoospermic controls (Cn; *n* = 17; BMI: 24.6 ± 2.0) and obese (Ob; *n* = 17; BMI: 32.6 ± 4.4) men. To determine the inflammation levels, we measured IL-6, TNF-α, and monocyte chemoattractant protein-1 (MCP1) by Magnetic Luminex^®^ Assay. mRNA expression of IL6, TNF-α, and hepcidin (HAMP) in PBMC were evaluated by RT-qPCR. The analysis of microRNAs was performed using the Taqman^®^ assays. The iron content in PBMC, seminal plasma, and spermatozoa was determined by Inductively Coupled Plasma Mass Spectrometry (ICP-MS). High serum IL6, TNF-α, and MCP1 levels were observed in Ob group (*p* < 0.05). Gene expression analysis showed an increased abundance relative of TNF-α (*p* = 0.018), HAMP (*p* = 0.03), and IL6 (*p* = 0.02) in PBMC from obese subjects. Also, we observed high levels of serum ferritin (*p* = 0.03), iron content in seminal plasma (*p* = 0.04), and spermatozoa (*p* = 0.002), but lower serum Fe (*p* = 0.007) in obese subjects. In the Ob group, a high expression of miR-155 (*p* = 0.02) and miR-21 (*p* = 0.03) was observed in PBMC and miR-122 (*p* = 0.03) in plasma. In sperm, both miR-155 (*p* = 0.004) and miR-122 (*p* = 0.028) were high in the Ob group. Our results showed that obese subjects have increased expressions of miR-155 and miR-122, two microRNAs that were previously related with inflammation and iron metabolism, respectively, at both the systemic and sperm levels.

## Introduction

Obesity is a worldwide pandemic with financial implications for both developed and developing countries (Wang Y. C. et al., 2011; Ng M. et al., 2014). This condition is a product of sustained positive energy balance, which leads to an increase in body weight. Increased availability of calorically dense foods, specifically an increase in fat and carbohydrate content, together with low physical activity or sedentary behavior are also associated with the development of overweight and obesity (Swinburn et al., 2011).

Inflammation plays a pivotal role in the presentation of cancer, gallbladder disease, stroke, cardiovascular disease, disability, hypertension, diabetes mellitus, osteoarthritis, and sleep apnea (Smith and Smith, 2016). Inflammation also relates to hypoxia, which can be explained by the increased adipose cell size in obese subject (Brook et al., 1972) and angiogenesis, which becomes rate limiting for adipose tissue expansion (Apovian et al., 2008). Both conditions limit oxygen diffusion, triggering necrotic processes in adipocytes (Brakenhielm et al., 2004), which stimulates macrophage infiltration to remove debris (Cinti et al., 2005) and produces pro-inflammatory cytokines such as IL6, TNF-α, and monocyte chemoattractant protein-1 (MCP1) (Hotamisligil, 2006). Inflammation is also related to anemia (Roy, 2010). Pro-inflammatory cytokines induce hepcidin (Hpc) expression, an iron hormone that reduces systemic iron levels (Nemeth et al., 2003). Obese subjects show lower serum iron and higher ferritin concentration, compared with normal-weight subjects. This condition is reversed when individuals lose weight.

MicroRNAs (miRNAs) are single-stranded non-coding RNA molecules of 19 to 24 nucleotides that act at post-translational level binding 3′UTR in messenger RNA (mRNA) inducing instability or degradation, which affects translation processes (Ha and Kim, 2014). Each microRNA represses multiple gene targets and a target mRNA can be repressed by multiple microRNAs (Felekkis et al., 2010). They play an essential role in many processes such as proliferation, development, differentiation, metabolism, and apoptosis (Bartel, 2004). Many miRNAs related to inflammation as miR-21 and miR155 have been previously described (Roy and Sen, 2011).

The gene pri-miR-21 located on chromosome 17 overlaps with the protein coding gene, TMEM 49, within their intronic region and it has an independent promoter region (Cai et al., 2004). IL6 induces miR-21 through the binding of STAT3 to regulatory region (Loffler et al., 2007). Phosphatase and tensin homolog (PTEN) is a target of miR-21 (Sheedy et al., 2010), which activates the transcription factor NF-κB. It regulates the expression of many genes, most of which encode cytokines, such as IL6 (Matsusaka et al., 1993). The miR-155 gene is located on chromosome 21 and is processed from an exon of a non-coding RNA transcribed from the B-cell Integration Cluster (BIC) (Zhang et al., 2008). Cai et al. (2012) demonstrated that miR-155 promotes the M1 phenotype of macrophage polarization. It produces large amounts of pro-inflammatory cytokines, such as TNF-α, IL6, and MCP1 (Lumeng et al., 2007). Inflammatory mediators like TNF-α induce miR-155 in macrophages and monocytes (O’Connell et al., 2007); therefore, there is a positive feedback response between TNF-α and miR-155.

MiRNA has also been implicated in the control of iron metabolism (Castoldi and Muckenthaler, 2012; Davis and Clarke, 2013). Iron increase triggers the bone morphogenetic protein (BMP)/SMAD signaling cascade via interaction with hemojuvelin (HJV) to active Hpc mRNA expression. Human hemochromatosis protein (HFE) and HJV are directly targeted by miR-122, thus decreasing hepcidin gene (HAMP) transcription (Castoldi et al., 2011). Ferritin is an iron-storage protein with the ability to accumulate large deposits of non-heme iron. Animal ferritins (Ft) are generally composed of two type of subunits, H for heavy and L for light. Only the H chain contains a ferroxidase center (Bradley et al., 2016), which is necessary for iron deposition. FtL facilitates iron nucleation and increases the turnover of the ferroxidase site. The FtH 3′UTR has a binding sequence to miR-200b. One study observed that increased FtH levels are correlated with decrease expression of this miR (Shpyleva et al., 2011).

Based in the previous information, we hypothesize that men with obesity show an increase in the relative expression of microRNAs associated with inflammation (miR-155 and miR-21) and iron homeostasis (miR-122 and miR-200b) at a systemic and spermatic level. Then, in the current study, we evaluated changes in microRNAs expression related to inflammation (miR-21 and miR155) and iron homeostasis (miR-122 and miR-200b) in peripheral blood mononuclear cells (PBMC), plasma, and spermatozoa of obese and healthy subjects. This transmission of miRNA patterns may be involved in the development of obesity in subsequent generations.

## Materials and Methods

This protocol was approved by the Ethical Review Board of the Institute of Nutrition and Food Technology (INTA) and the Central Metropolitan Health Service, Santiago, Chile. All subjects read, and signed an informed consent. We studied 17 normal weight individuals (BMI 20–26 kg/m^2^; Cn group) and 17 obese patients (BMI ≥ 30 kg/m^2^; OB group), who received care at either the Institute of Maternal and Child Research, San Borja Clinical Hospital or at INTA. As inclusion criteria, all individuals were normozoospermic volunteers in reproductive age (25–40 years). We excluded from the study subjects who had fasting blood glucose >200 mg/dL; subjects who consumed more than 10 g of tobacco per day; subjects who reported diseases in the 15 days prior to sampling; who used treatment to lose weight or who consumed some supplement of iron (at least the 6 months previous to the study). To determine body mass index (BMI), all patients were weighed and measured. A sample of 35 mL of blood was obtained from the antecubital vein after an overnight fast.

A total of 8 mL of blood was used to measure biochemical indicators such as: glycemia (by glucose oxidase reaction; Dialab, Austria), insulin (by radioimmunoassay; Coat-A-Count, Siemens, LA, United States), lipid profile (by colorimetric method), and hsCRP (by immune precipitation in liquid phase; Orion Diagnostica, Espoo, Finland). IL6, TNF-α, and MCP1 were determined by Magnetic Luminex^®^ Assay (R&D Systems, Minneapolis, United States). A total of 3 mL of blood was used to measure hematological and iron nutritional status. Hemoglobin and hematocrit were measured in a Counter Cell Dyn 1700. Serum ferritin by ELISA (Cook, 1985) and serum iron by atomic absorption spectrometry with graphite furnace (Simaa 6100, Perkin Elmer), using as internal controls MR-CCHEN-002 (Venus antiqua) and DOlt-2 (Dogfish liver) and 5-point calibration curve using an iron standard of 1,000 μg/L were used (Merck, Germany No. 1.19781.0500).

A totla of 24 mL of blood samples was collected with EDTA anticoagulant to isolate PBMCs using Histopaque-1077 (Sigma, St. Louis, MO).

For sperm studies, a single ejaculation was collected from participants, who were requested of sexual abstinence for at least 2–5 days prior. Semen samples were obtained by masturbation into a sterile plastic specimen container. Samples were incubated at 37°C for 30 min and conventional semen parameters were analyzed according to the World Health Organization (2010) guidelines. Non-spermatic cells were excluded by differential lysis according to Jeffreys et al. (1994).

### miRNA and mRNA Expression

Total RNA was isolated from PBMCs and spermatozoa using Trizol reagent (Invitrogen) following manufacturer protocol. RNA concentrations and purity were determined using Biowave II UV/Visible Spectrophotometer (Biochrom).

The miRNA-21, miRNA-155, miRNA-122, and miRNA-200b expression were measured using TaqMan^®^ miRNA Reverse Transcription Kit and Taqman^®^ single miRNA assays (Applied Biosystems, Life Technologies). U6 was used as a housekeeping gene miRNA for normalization. All qPCR reactions were carried out in triplicate. The StepOne Plus (Applied Biosystems) was used for real-time PCR using TaqMan^®^ Universal Master Mix II, and does not contain Uracil *N*-glycosylase (UNG) (Applied Biosystems). Relative quantification of the expression levels used the comparative threshold cycle (ΔCt) method (Livak and Schmittgen, 2001).

For mRNA expression, 1 μg RNA was transcribed into cDNA using a high-capacity cDNA reverse transcription kit for mRNA (Applied Biosystems). Beta-2-microglobulin (B2M) was used as a housekeeping gene. The primers used (**Table 1**) for B2M, hepcidin, IL6, and TNF-α were described by Andrews et al. (2015). Real Time PCR was performed using Fast SYBR^®^ Green Master Mix on Step One Plus systems (Applied Biosystems). Melting curve analysis was constructed to verify the presence of gene-specific amplification and for absence of primer dimers. Final results were reported according to the Pfaffl (2001) method.

**Table 1:** Primers for quantitative real-time RT-PCR.

Primer	Sequence	Fragment length (bp)
BM2 forward	GAT GCC GCA TTT GGA TTG GA	187
BM2 reverse	TGG AGC AAC CTG CTC AGA TA	
TNFA forward	GTT CCT CAG CCT CTT CTC CT	186
TNFA reverse	ACA ACA TGG GCT ACA GGC TT	
Hpc forward	GAC ACC AGA GCA AGC TCA A	134
Hpc reverse	GAA AAC AGA GCC ACT GGT CA	
IL6 forward	ATG TCT GAG GCT CAT TCT GC	198
IL6 reverse	GCG GCT ACA TCT TTG GAA TC	

### Iron Levels in PBMCs, Spermatozoa, and Seminal Plasma

Iron levels in PBMCs, spermatozoa, and seminal plasma samples were analyzed by means of inductively coupled plasma mass spectrometry (ICP-MS, Agilent 7700, Tokyo, Japan) equipped with a He collision cell. Previously, PBMCs and spermatozoa were washed twice with water obtained by a Milli-Q system. Calibration curves were prepared following the Ga addition technique as an internal standard, using stock solutions of 1,000 mg/L of each element (Merck).

Samples were diluted with a solution containing 2% 1-buthanol, 0.05% EDTA, 0.05% Triton X-100, and 0.5% NH4OH. Calibration curves were prepared under the same conditions. The accuracy of the method was evaluated by the analysis of a suitable certified reference material, Seronorm (Billingstad, Norway), and by recovery studies in samples enriched with an iron standard. The calculated recoveries were between 95% and 105% in all cases.

### Statistical Analysis

The sample size was determined with the statistical Infostat program (Di Rienzo et al., 2011). We used a difference of 1 unit in the relative expression of microRNAs, with 1 standard deviation in each group and a power of 80%. Differences in anthropometric, semen, and biochemical parameters, iron measurement and gene expression among control and obese groups were evaluated using the Mann–Whitney test. The association between variables was performed with rho Spearman. Data were presented as median and interquartile range. Statistical significance was assigned as *p* < 0.05. Statistical analysis was performed using GraphPad Prism 6.0 software.

## Results

### Anthropometry and Biochemical Parameters

Anthropometric measurements such as weight, BMI, and waist circumference were higher in the Ob compared to Cn (**Table 2**). No differences were found in the blood lipid profile and transaminase values between groups. Obese subjects had high values of basal glycemia, basal insulin, and HOMA-IR, indicating insulin resistance in this group (**Table 2**).

**Table 2:** Anthropometrical and biochemical features of participants.

Characteristics	Cn	Ob	*P*-value^∗^
*n*	17	17	
Age (year)	30 (26.5–36)	31 (28.5–38.5)	0.1
Weigh (Kg)	74.8 (70.8–77.9)	98.8 (88.5–108.3)	<0.0001
BMI (kg/m^2^)	24.6 (24.0–26.0)	32.6 (30.9–35.3)	<0.0001
Abdominal circumference (cm)	83.5 (79.3–90.2)	114 (100–115)	<0.0001
Total Cholesterol (mg/dL)	178 (153–215)	178 (157–190)	0.30
HDL-C (mg/dL)	40.4 (27.9–47.0)	35 (29.7–40.6)	0.20
LDL-C (mg/dL)	118.2 (92.2–155.6)	107 (94.66–123.7)	0.20
Triglycerides (mg/dL)	134.6 (80.8–175.3)	133 (94.6–189.5)	0.10
GPT (mg/dL)	28.2 (17.7–42.7)	33.6 (22.3–45.3)	0.10
GGT (mg/dL)	37.5 (23.6–55)	45.3 (34.6–56.2)	0.10
GOT (mg/dL)	31.8 (25.4–41.5)	40.3 (29.8–59.3)	0.05
Basal Glycemia (mg/dL)	95.2 (84.3–101.3)	98.3 (92.9–110.5)	0.03
Basal Insulin (ng/mL)	4.7 (1.9–8.6)	13.9 (8.2–22.4)	0.0005
Glycosylated hemoglobin AC1 (%)	4.2 (3.6–5.4)	5.2 (3.8–6.5)	0.10
HOMA-IR	1.2 (0.4–1.9)	3.4 (1.8–6.1)	0.0006

*BMI, Body mass index; HDL-C, High density lipoprotein-cholesterol; LDL-C, Low density lipoprotein-cholesterol; GPT, glutamate Pyruvate Transaminase; GGT, Gamma-Glutamyl Transferase; GOT, Glutamate Oxaloacetate Transaminase. Values are median and range interquartile. ^∗^Mann–Whitney test.*

Iron nutrition parameters showed that hematocrit and hemoglobin were similar between Ob and Cn subjects (*p* = NS). However, serum iron and ferritin were different between Ob and Cn subjects (*p* < 0.007 and *p* < 0.03, respectively) (**Table 3** and **Figure 5**). Also, serum IL-6 (*p* < 0.02), TNF-α (*p* < 0.03), and MCP1 (*p* < 0.03) levels were higher in the obese group than controls subjects (**Table 3**).

**Table 3:** Parameters of iron nutrition and inflammation.

Characteristics	Cn	Ob	*P*-value^∗^
*n*	17	17	
Hemoglobin (g/dL)	16.4 (15.4–17)	17 (16.1–17.6)	0.08
Hematocrit (%)	46.3 (44.5–49.2)	48.8 (46.4–49.7)	0.07
Serum Iron (μg/dL)	151 (115–166)	107 (81–128)	0.007
Serum Ferritin (μg/L)	49.5 (43.5–68.6)	67.7 (56.3–85.8)	0.03
hsCRP (mg/L)	0.9 (0.9–1.4)	1.0 (0.8–1.6)	0.40
IL-6 (pg/mL)	0.6 (0.5–0.7)	0.9 (0.7–1)	0.02
TNF-α (pg/mL)	1.1 (1.1–1.2)	1.9 (1.2–2.55)	0.03
MCP1 (pg/mL)	82 (73.1–134)	140 (107.8–163.8)	0.03

*hsCRP, high sensitivity C-reactive protein. Values are median and range interquartile. ^∗^Mann–Whitney test.*

In relation to sperm quality parameters, significant statistical differences were found in the analysis of progressive sperm motility, immotile sperm, and tail defects (**Table 4**), which accounts for the effects of obesity on motility spermatic.

**Table 4:** Semen parameters of participants.

Semen parameters	Cn	Ob	*P*-value^∗^
*n*	17	17	
Abstinence time [days]	4.0 (3.0–4.5)	5.0 (2.5–5.0)	0.30
Semen pH	8.0	8.0	0.70
Semen volume [mL]	3.0 (2.0–4.7)	2.0 (1.6–2.9)	0.06
Sperm concentration [10^6^/mL]	86 (54.5–200)	78 (29.5–200)	0.20
Sperm count [million]	312 (125–506)	226 (47–485)	0.10
Sperm progressive motility [%]	61 (58–64)	55 (50.5–60.5)	0.007
Sperm non-progressive motility [%]	4 (2.5–5)	4 (2.5–5)	0.40
Sperm immotility [%]	34 (31.5–38.5)	40 (35.5–46.5)	0.01
Sperm vitality [%]	83 (77.5–89)	80 (71.5–85)	0.10
Krugger’s morphology [%]	6.0 (5.0–7.0)	5.5 (4.2–8.8)	0.80
Head defects [%]	99 (98–100)	99 (98–100)	0.90
Mid-piece defects [%]	44 (29–48)	31 (24–39)	0.10
Tail defects [%]	5.0 (3.0–7.4)	8.2 (5.7–15.7)	0.02

*Values are median and range interquartile. ^∗^Mann–Whitney test.*

### Relative Abundance of Genes Related to Inflammation and Iron Metabolism

**FIGURE 1:**
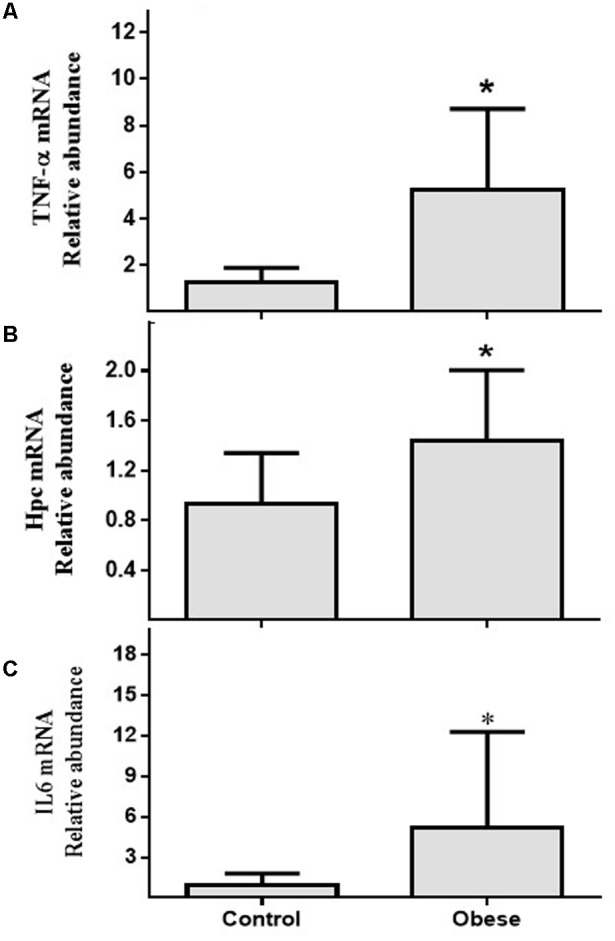
mRNA relative abundance of genes related to inflammation and Fe metabolism in PBMCs. **(A)** TNF-α (*p* = 0.018); **(B)** Hpc (*p* = 0.03); **(C)** IL6 (*p* = 0.02). Values are median and range interquartile. Mann–Whitney test. ^∗^Indicates significant difference.

In PBMCs, there was significant differences in the relative mRNA abundance of TNF-α (**Figure 1A**), HAMP (**Figure 1B**), and IL6 (**Figure 1C**) between Ob and Cn (*p* = 0.018; *p* = 0.019 and *p* = 0.02, respectively).

**FIGURE 2:**
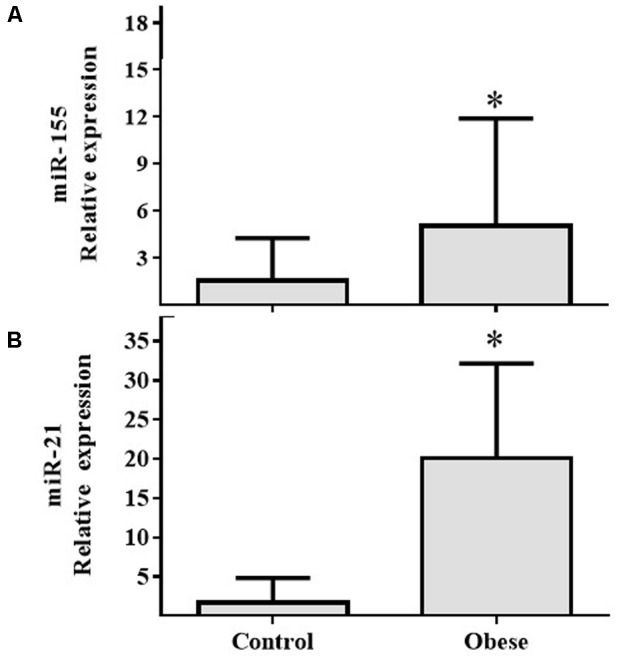
miR-155 and miR-21 levels in PBMCs of the obese subjects compared with the normal weight controls. **(A)** Differential relative miR-155 expression levels verified by qRT-PCR in the serum samples from normal weight (*n* = 17) and obese (*n* = 17) subjects (*p* = 0.025). **(B)** Differential relative miR-21 expression levels in the serum samples from normal weight (*n* = 17) and obese (*n* = 17) subjects (*p* = 0.031). Values are median and range interquartile. Mann–Whitney test. ^∗^Indicates significant difference.

**FIGURE 3:**
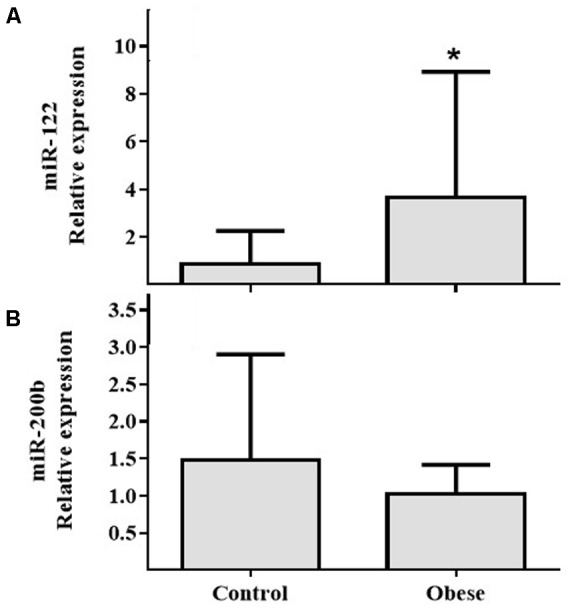
The circulating miR-122 and miR-200b levels in the obese subjects compared with the normal weight controls. **(A)** Differential relative miR-122 expression levels verified by qRT-PCR in the serum samples from normal weight (*n* = 17) and obese (*n* = 17) subjects (*p* = 0.03). **(B)** Differential relative miR-200b expression levels in the serum samples from normal weight (*n* = 17) and obese (*n* = 17) subjects (*p* = NS). Values are median and range interquartile. Mann–Whitney test. ^∗^Indicates significant difference.

In PBMCs, expressions of miR-155 (**Figure 2A**) and miR-21 (**Figure 2B**) showed increased expression in Ob compared with Cn (*p* = 0.025 and *p* = 0.031, respectively). In plasma, miR-122 levels were increased in Ob group (*p* = 0.029; **Figure 3A**); however, miR-200b did not show a significant difference (*p* = 0.059; **Figure 3B**) between obese and control subjects.

**FIGURE 4:**
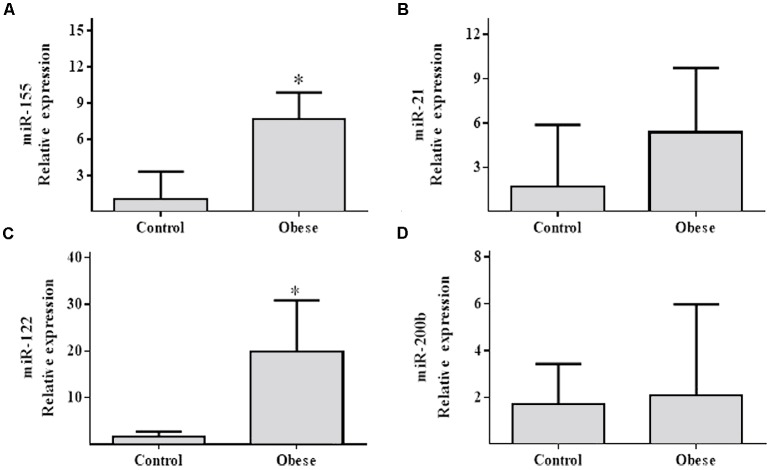
Relative expression of miR-155, miR-21, miR-122 and miR-200b in the spermatozoa samples of obese subjects compared with the normal weight controls. **(A)** Differential relative miR-155 expression levels verified by qRT-PCR in the serum samples from normal weight (*n* = 17) and obese (*n* = 17) subjects (*p* = 0.00477). **(B)** Differential relative miR-21 expression levels (*p* = NS). **(C)** Relative expression of miR-122 (*p* = 0.028). **(D)** Relative expression of miR-200b (*p* = NS). Values are median and range interquartile. Mann–Whitney test. ^∗^Indicates significant difference.

In spermatozoa samples, miR-155 and miR-122 levels were elevated in Ob (*p* = 0.005; *p* = 0.028, respectively) (**Figures 4A,C**) compared to Cn. However, miR-21 and miR-200b showed no significant differences (*p* > NS; **Figures 4B,D**).

**FIGURE 5:**
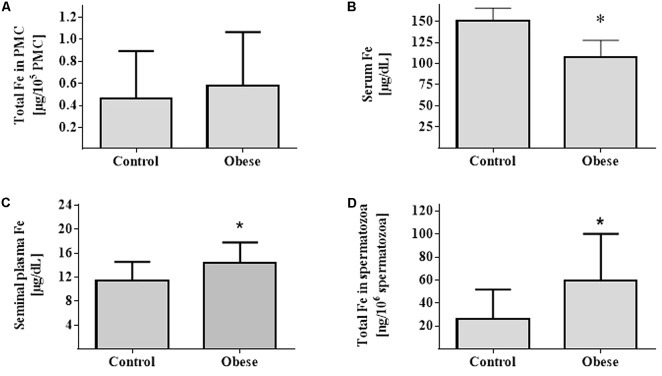
Measurement of Iron. Fe content in PBMCs **(A)**, serum **(B)**, seminal plasma **(C)**, and spermatozoa **(D)** (Control group; *n* = 17) (Obese group; *n* = 17). Values are median and range interquartile. Mann–Whitney test. ^∗^Indicates significant difference.

We observed an association between miR-155 (rho Spearman = 0.41; *p* = 0.02) and mIR-21 (rho Spearman = 0.39; *p* = 0.02) with the relative expression of TNF-α mRNA in PBMCs. In sperm, miR-155 (rho Spearman = 0.84; *p* = 0.002) and miR-21 (rho Spearman = 0.71; *p* = 0.03) with serum IL-6. Also, we observed an inverse association between serum iron levels and the expression of miR-122, considering the total population (controls and obese subjects; rho Spearman = -0.54; *p* = 0.02)

### Iron Content in PBMCs, Spermatozoa, and Seminal Plasma

Iron content in PBMCs in controls was 0.46 (0.14–0.89) μg/10^5^ cells compared to 0.57 (0.16–1.06) μg/10^5^ cells in obese subjects (*p* = NS). Spermatozoa showed an iron content of 26.3 (8.1–51.6) ng/10^6^ cells and 59.7 (27.5–118.2) ng/10^6^ cells in Cn versus Ob, respectively (*p* = 0.002). In seminal plasma, the iron content in Cn was 11.5 (9.2–14.6) μg/dL versus 14.1 (10.9–17.8) μg/dL in Ob (*p* = 0.04).

## Discussion

Obesity is a public health problem and different strategies have been implemented for the treatment of this pathology, which have not yielded the expected results. Thus, determining early markers of obesity development, even before conception, can be important to understand and prevent the occurrence. Previous literature has demonstrated that circulating microRNAs serve as clinical biomarkers, as they are quantifiable and sufficiently stable (Chen et al., 2008; Mitchell et al., 2008). The present work evaluated the expression of microRNAs related to regulation of inflammation and iron homeostasis at the systemic and spermatozoa level, which may be of interest as sperm cells transmit genetic information to offspring.

In this work, differences in parameters related to sperm motility were observed, but not in sperm counts. Previous work has reported an effect of BMI in total sperm count and sperm concentration (Jensen et al., 2004; Qin et al., 2007; Aggerholm et al., 2008). Kort et al. (2006) demonstrated a negative association between BMI and the total number of normal-motile sperm cells. A study with a large patient sample size (*n* = 10,000), found a negative correlation: increased BMI was inversely associated with decreased seminal volume, sperm concentration, and sperm motility (Belloc et al., 2014).

As expected, in the present study we found statistically significant differences between normal weight and obese subjects in anthropometrical parameters such as body weight, BMI, and waist circumference, along with differences in parameters related to glycemic control being indicative of a condition of insulin resistance characteristic of obesity. Regarding inflammation and iron-related parameters, obese patients did not present with anemia associated with inflammation, but an increase in serum ferritin levels were found in obese patients with respect to the control group, in accordance with our previous studies (Andrews et al., 2015). Ferritin is used as a marker of iron deficiency at the clinical level (Gupta Bansal et al., 2015). As an acute phase protein, serum ferritin level tends to be higher in people with BMI ≥25 kg/m^2^ due to a subclinical inflammatory state (Huang et al., 2015; Kim et al., 2015). Thus, serum ferritin is not an adequate marker of iron deficiency or iron deficiency anemia in overweight and obese persons.

Previous studies demonstrated that obese subjects have higher circulating levels of IL6, TNF-α, and hepcidin (Hotamisligil, 2006; Amato et al., 2010). In accordance, this work found higher serum IL6 and TNF-α levels and hepcidin, IL6, and TNF-α mRNAs expression in PBMC in obese subjects compared to controls. The small peptide hormone hepcidin controls the amount of iron available in circulation by interacting with the iron exporter ferroportin (Ganz and Nemeth, 2012). This hormone is predominantly expressed by the liver, although other tissues such as heart, brain, and monocytes can express it at low levels (Zhang and Rovin, 2013). Hepcidin levels are controlled by three hepatic signaling pathways: the HH-associated proteins such as HFE/TfR2 (transferrin receptor 2); HJV/Bmp/Smad signaling pathway, which controls hepcidin response to systemic iron availability; and IL6/Jak/Stat pathway, which control HAMP expression in the inflammatory response (Schmidt, 2015).

MiR-122 is highly abundant in liver tissue and is a hepato-specific miRNA (Wrighting and Andrews, 2006). The liver specific miR-122 directly targets HFE and HJV, contributing to the regulation of systemic iron homeostasis by decreasing hepcidin mRNA expression (Chen et al., 2008; Mitchell et al., 2008). MiR-122 inhibition increases the amount of mRNA transcribed by genes that control systemic iron levels, such as HFE, HJV, bone morphogenetic protein receptor type 1A (Bmpr1a), and HAMP (Castoldi and Muckenthaler, 2012). Li et al. (2017) and Tang et al. (2017) have demonstrated that iron overload in mice induces the down-regulation of miR-122. Also, in patients with iron overload disorders, it was observed that miR-122 was decreased. Iron overload induces a significantly reduced expression of miR-122, which also increases HFE and HJV expression (Castoldi et al., 2011).

We found that plasma and sperm miR-122 concentration was higher in obese compared to control subjects. These results coincide with previous studies that demonstrate that circulating miR-122 is elevated in obese patients and exhibit a tendency to increase with the degree of obesity (Ortega et al., 2013; Wang et al., 2015). We propose that an increase in the expression of cytokine IL-6 stimulates the expression of ferritin and hepcidin, decreasing serum iron levels, which is a stimulus to increase the expression of miR-122. This would act by inhibiting HFE and HJV pathway, to reduce HAMP expression. Other mechanism that may be involved in the up-regulate of miR-122 in obese subjects involves NF-κB activity. In the promoter region of miR-122 was identified a NF-κB binding site, and has been demonstrated that RELA (NF-κB p65 subunit), is an activator of NF-κB, which increased promoter activity of miR-122 (Rivkin et al., 2016).

Donkin et al. (2016) did not find changes in expression profiles of miR-122 at the spermatic level, by contrast, our data demonstrated that miR-122 in spermatozoa was elevated among obese patients. This difference can be explained by the treatment of the sample, Donkin et al. (2016) performed a “swim-up” procedure to exclude somatic cells and to isolate motile spermatozoa, while this study only excluded somatic cells, including immotile and motile spermatozoa to analysis of miRNA expression. Wang C. et al. (2011) studied miRNAs in seminal plasma and observed an increase of miR-122 expression in asthenozoospermic patients, a term for reduced sperm motility. In contrast, Abu-Halima et al. (2013) found that miR-122 was down regulated in sperm from asthenozoospermic and oligoasthenospermic infertile men, compared to normozoospermic controls. In this study, the increase of miR-122 was related to the obesity and not to differences in the seminal pattern between cases and control subjects. However, our results seem to show an association with the sperm motility and tail morphology of the sperms. To study the real significance of these findings will require greater and more detailed studies. We did not find any significant correlation between miR-122 and iron in sperm or seminal plasma. We only observed an inverse association between serum iron levels and the expression of serum miR-122, considering the total population (controls and obese; rho Spearman = -0.54, *p* = 0.02).

MiR-155 is a component of the primary macrophage response to different types of inflammatory mediators such as TNF-α, which can induce miR-155 in macrophages and monocytes (O’Connell et al., 2007). On the other hand, up-regulation of miR-155 enhances TNF-α production. It has been hypothesized that miR-155 could directly increase TNF-α levels by augmenting transcript stability through binding to its 3′UTR. miR-155 could also target gene transcripts coding for proteins that are known to be repressor of TNF-α translation (Faraoni et al., 2009). Karkeni et al. (2016) demonstrated in 3T3L1 adipocytes (mouse) and human adipose tissue that miR-155 is induced by inflammation and that miR-155, in turn, participates in the overall effect of TNF-α, and more globally in the amplification of the inflammatory phenotype. We found that PBMCs and spermatozoa miR-155 expression increased in obese patients with respect to control subjects.

Mahdavi et al. (2018) showed a decreased expression of miR-155 in serum of obese subjects, which was found to be related to BMI. Also, Marques-Rocha et al. (2016) agree with our results. They observed that obese subjects who underwent a nutritional intervention, reduce their anthropometric parameters and decrease the expression of miR-155 in white blood cells after 8 weeks of treatment. These results are in agreement with those found Donkin et al. (2016), who observed an increase in the expression of this microRNA in spermatozoa. There is evidence that miR-155 is relevant in the development of obesity. Studies in miR-155 knock out female mice fed with a high-fat diet showed a phenotype with less fat and body-weight than wild type mice. In addition, miR-115 increases adipogenic, insulin sensitivity, and energy uncoupling machinery, while limiting inflammation in white adipose tissue, which together could restrict high-fat diet-induced fat accumulation (Gaudet et al., 2016).

Iron is an important trace metal, vital for cell growth and development, which participates in oxygenation and reduction processes and acts as a cofactor for many enzymes. Animal studies have demonstrated that high testicular iron concentration is correlated with small size of testes (Wise et al., 2003). In addition, a significant increase in the iron level of seminal plasma correlated with oxidative damage in sub fertile men (Aydemir et al., 2006). Differences in iron plasma seminal levels have been reported in sub fertile subjects (Massaìnyi et al., 2004; Aydemir et al., 2006; Marzec-Wróblewska et al., 2011). Others studies have demonstrated that iron can affect negatively the morphology of spermatozoa (Massaìnyi et al., 2004). Huang et al. (2001) observed that incubation with Fe^2+^ caused a reduction of sperm motility, associated with a marked lipid peroxidation. In the present work, we found differences in iron concentration between control and obese group: lower in serum and higher in seminal plasma and spermatozoa from obese subject. Obesity is characterized by mild chronic inflammation; this would increase serum hepcidin levels, which acts iron redistributing from extracellular spaces. We speculate that the process of inflammation produces an accumulation of iron in the testicle and in organs such as prostate, seminal vesicle, and bulbourethral glandule. Also, during the process of spermatogenesis, at the testicular level and during the process of maturation in the epididymis, through the epididymosomes (Sullivan, 2015), iron would be incorporated to the sperm and seminal plasma.

On the other hand, in 1952, Chile implemented the program of fortification of wheat flour with iron with the aim of reducing the prevalence of iron-deficiency anemia in the population at greatest risk, such as children, pregnant women, and children of childbearing age (Mujica et al., 2012). However, this increase in iron availability is also reaching the adult male population, which is not at risk of anemia. On the other side, the RDA for iron at this age is 7.7 mg/day, however, in Chile the intake of iron is 15.5 mg/day, according to the last National Survey of Food Consumption (Ministerio de Salud de Chile [MINSAL], 2010). Given that previous studies have shown that there is an alteration in iron homeostasis in obese subjects. We were interested in studying in the Chilean population what happens with this micronutrient.

Epidemiological studies in humans indicate that the nutritional status of parents predisposes offspring to a greater or lesser risk in the development of obesity and cardiovascular diseases (Kaati et al., 2002; Pembrey, 2010). Studies in the murine model have showed that male rats consuming a high-fat diet predispose their female offspring to dysfunction of pancreatic β cells, accompanied by an increase in body weight, adiposity, glucose intolerance, and an alteration in insulin sensitivity (Carone et al., 2010; Ng et al., 2010; Ng S.F. et al., 2014). These results suggest that molecular mechanisms are being transmitted in sperm. These microRNAs have been detected in mature sperm, which could be in contact with the oocyte during the fertilization (Ostermeier et al., 2004; McPherson et al., 2015).

How the expression of microRNA of sperm respond to environmental factors such as stress, diet or exercise and how these type of molecular mechanisms can be transmitted to the following generations have been studied in the murine model (Fullston et al., 2013; Gapp et al., 2014; McPherson et al., 2015; Short et al., 2016). Fullston et al. (2013) determined changes in mRNA expression related to the network of lipid metabolism and inflammation. We studied the miR-155, which has been previously reported to have a positive feedback with TNF-alpha. However, as is the case of other microRNAs, this miR has been shown to be related to adipose tissue in both mouse and human studies (Klöting et al., 2009; Chen et al., 2013; Gaudet et al., 2016). It has been established that miR-155 present a site binding to the region 3′UTR of the Liver X receptor alpha (LXR-alpha), which are nuclear receptors that widely modulate lipid metabolism (Wang et al., 2016). Also, miR-155 regulated the expression of CEBPβ and PPARγ in adipocytes (Liu et al., 2011; Chen et al., 2013) and miR-122 participate regulating the levels of cholesterol, fatty acids synthesis, and in the cell differentiation (Kim et al., 2011).

As some limitations of our study, we must consider our small sample size (34 male subjects). Additional, larger studies are necessary to confirm our results. We should enrich the evaluation of the nutritional iron status including hepcidin levels and transferrin saturation.

## Conclusion

Different studies have explored the association that microRNAs have with different pathologies such as cancer, type 2 diabetes mellitus, and obesity, among others. Our study demonstrated that two microRNAs, miR-122 and miR-155, are high in the plasma, PBMC, and spermatozoa from obese compared to control subjects. These findings are important as they may provide additional potential targets to prevent the development of obesity.

## Author Contributions

PL wrote the paper, performed the PCR assays, and contributed substantially to the design, statistical analyses, and interpretation of the results. AC contributed to the experimental design, direction of seminal analysis, and revision of the manuscript. MF performed the seminal analysis and contributed with clinical evaluations. KM performed the biochemical profiling. PA, CS-G, and JL contributed with iron measured by ICP-MS and to the revision of the manuscript. MA conceived the study, directed the experimental protocols and statistical analysis, and revised the manuscript.

## Conflict of Interest Statement

The authors declare that the research was conducted in the absence of any commercial or financial relationships that could be construed as a potential conflict of interest.
